# Supplementary material for: Force–reversible chemical reaction at ambient temperature for designing toughened dynamic covalent polymer networks
Source: Nat Commun. 2022 Jun 9;13:3231. doi: 10.1038/s41467-022-30972-7 (PMC9184613; doi:10.1038/s41467-022-30972-7)
Supplement: Supplementary file 1 — Supplementary Information [file 41467_2022_30972_MOESM1_ESM.pdf]

Supplementary Materials for

Force–Reversible Chemical Reaction at Ambient Temperature for Designing Toughened Dynamic Covalent Polymer Networks

Mengqi Du,[1] Hannes A. Houck,[2] Qiang Yin,[4] Yewei Xu,[1] Ying Huang,[1] Yang Lan,[5] Li Yang,[1,3]* Filip E. Du Prez,[2]* Guanjun Chang[1,3]*

*Corresponding authors.

Filip E. Du Prez, E-mail: filip.duprez@ugent.be;

Guanjun Chang, Email: gjchang@mail.ustc.edu.cn;

Li Yang, Email: yanglichem628@126.com.

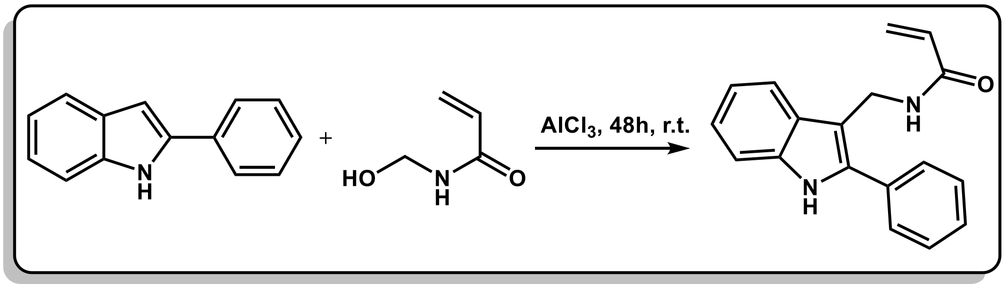

Supplementary Figure 1. Synthesis route of *N*-(1*H*-2-phenyl-indole-3-methyl) acrylamide (NPI) through electrophilic substitution reaction.

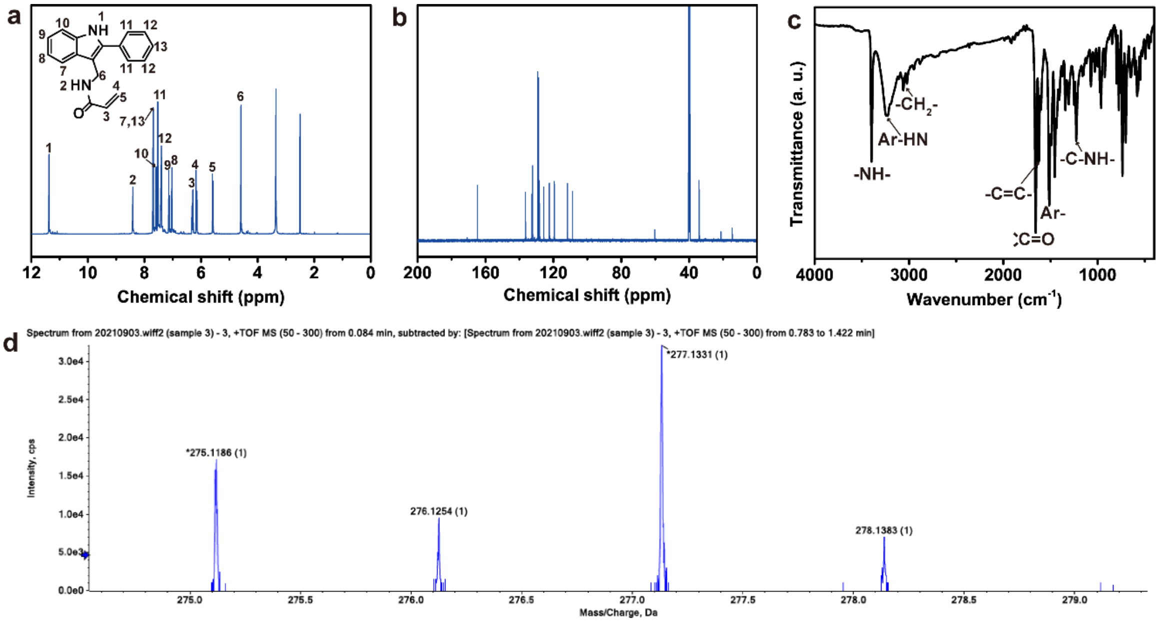

Supplementary Figure 2. a ^{1}H NMR, **b** ^{13}C NMR (in DMSO-d_6), **c** FT-IR and **d** HRMS of compound NPI.

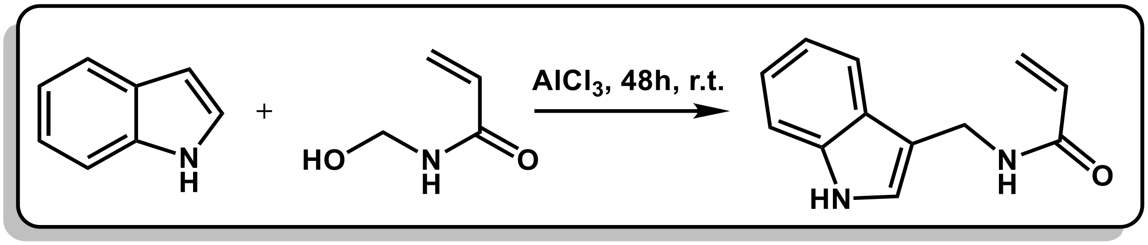

Supplementary Figure 3. Synthesis route of N-(1H-indole-3-methyl) acrylamide (NIAM) through electrophilic substitution reaction.

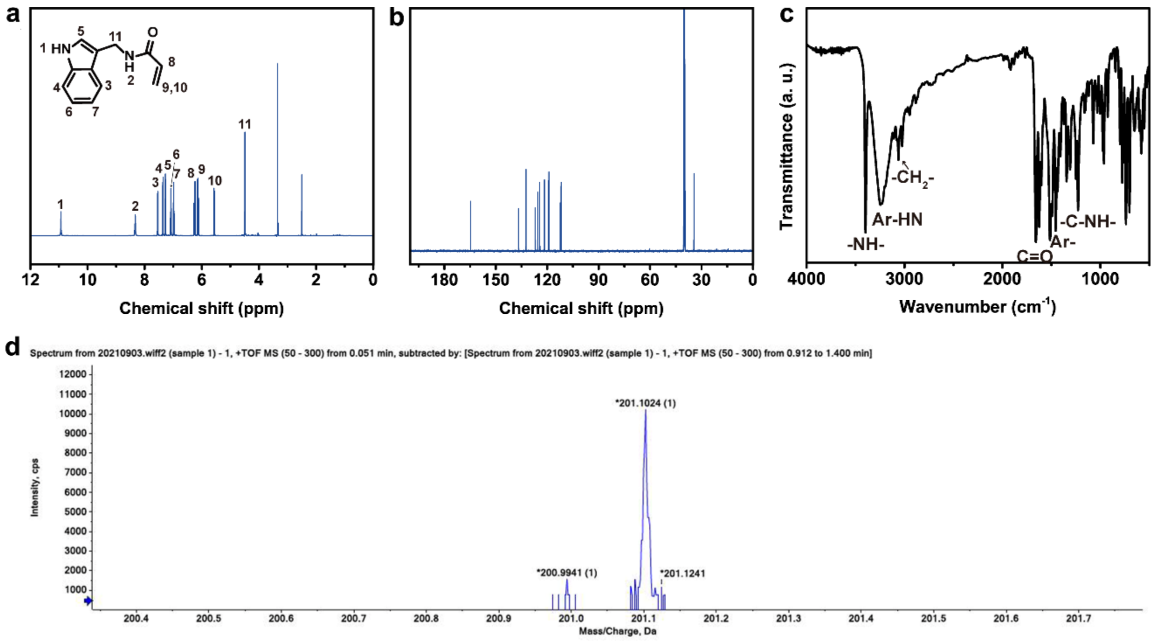

Supplementary Figure 4. a ^{1}H NMR, **b** ^{13}C NMR (in DMSO-*d*$_6$), **c** FT-IR and **d** HRMS of compound NIAM.

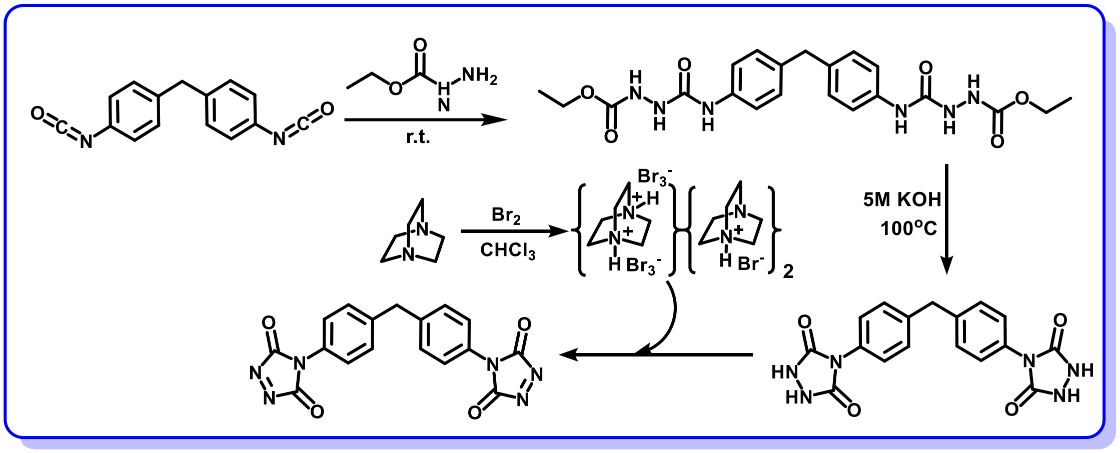

Supplementary Figure 5. The preparation route of 4,4’-(4,4’-diphenylmethylene)-bis-(1,2,4-triazoline-3,5-dione) (MDI-TAD).

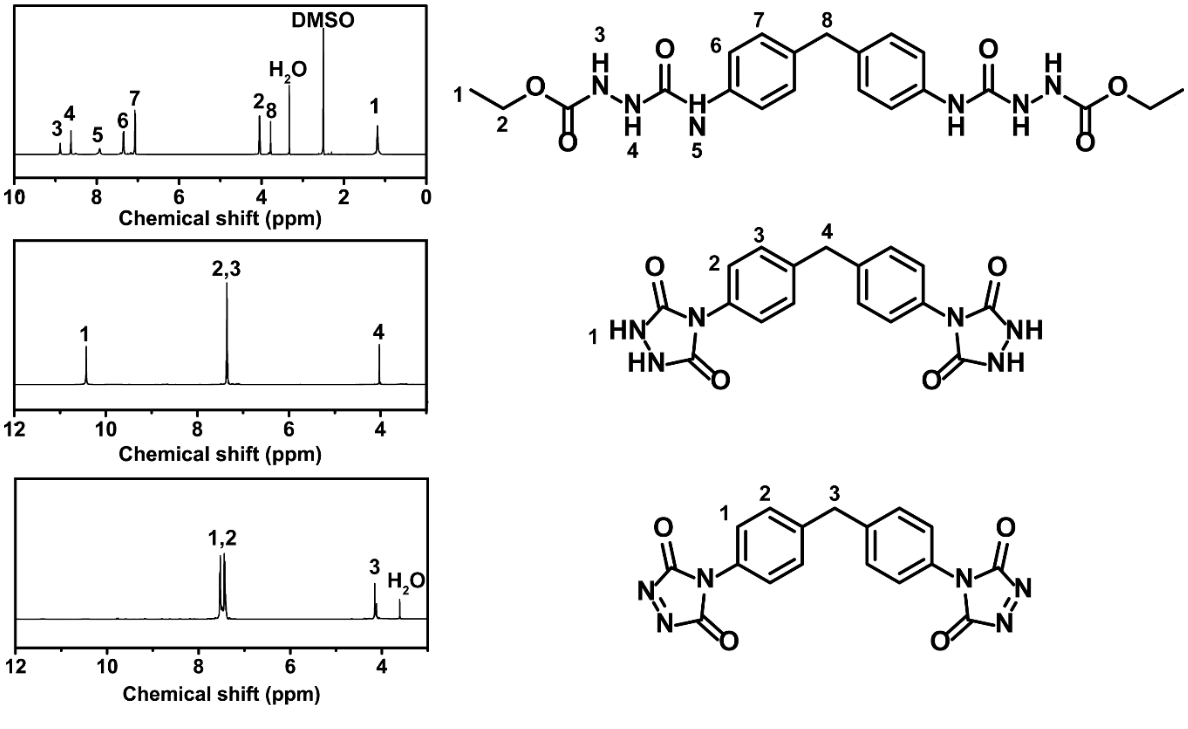

Supplementary Figure 6. ^{1}H NMR (in DMSO-*d*$_6$) of intermediate 4,4’-(4,4’-diphenylmethylene)-bis-(carbethoxysemicarbazide), 4,4’-(4,4’-diphenylmethylene)-bis-(urazole) (MDI-bisurazole), and the final 4,4’-(4,4’-diphenylmethylene)-bis-(1,2,4-triazoline-3,5-dione) (MDI-TAD) cross-linker.

Supplementary Table 1. Polymer network compositions. Monomer composition of reaction mixtures for each cured network. The mass of each polymer system is about 10 g as example.

**CPMA** - Network 1 - NPI : MA : MDI-TAD = 1 : 20 : 0.5				
	NPI	MA	MDI-TAD	
Mole (mmol)	5	100	2.5	
Mass (mg)	1400	8600	905	
Mass Percent (%)	12.8	78.8	8.4	
**ir-CPMA** - Network 2 - NIAM : MA : MDI-TAD = 1 : 20 : 0.5				
	NIAM	MA	MDI-TAD	
Mole (mmol)	5.21	104.2	2.6	
Mass (mg)	1042	8961	941	
Mass Percent (%)	9.5	81.9	8.6	
**du-CPMA** - Network 3 - NPI : NIAM : MA :MDI-TAD = 0.5 : 0.5 : 20 : 0.5				
	NPI	NIAM	MA	MDI-TAD
Mole (mmol)	2.55	2.55	102	2.55
Mass (mg)	704	510	8786	923
Mass Percent (%)	6.44	4.68	80.43	8.45
**CPMMA-** Network 4 - NPI : MMA : MDI-TAD = 1 : 20 : 0.5				
	NPI	MMA	MDI-TAD	
Mole (mmol)	4.4	87.8	2.2	
Mass (mg)	1220	8780	796	
Mass Percent (%)	11.3	81.3	7.4	

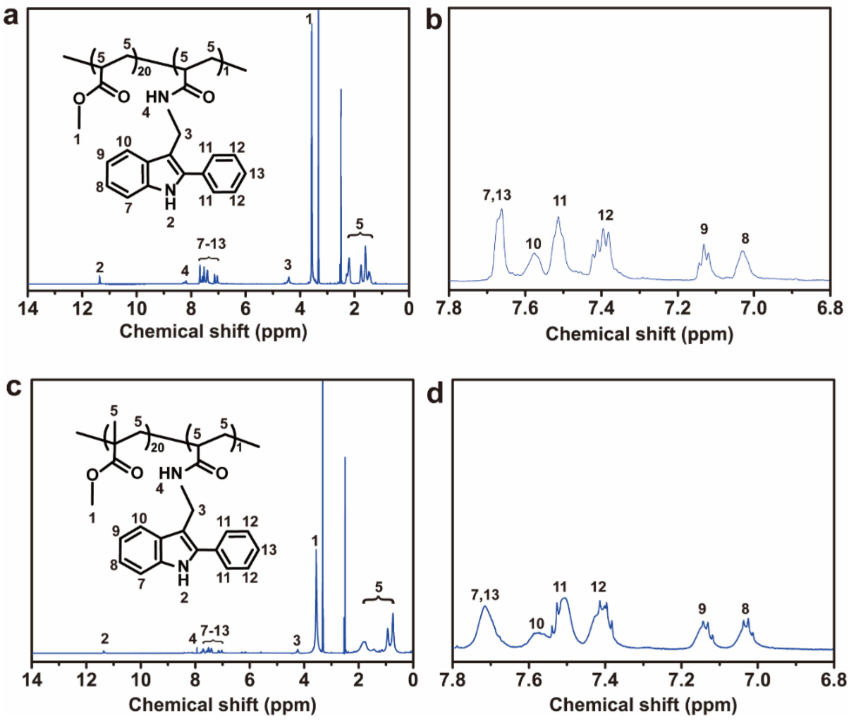

Supplementary Figure 7. a ^{1}H NMR (in DMSO-d_6) of CPMA and **b** its local enlarged drawing from a range of 7.8-6.8 ppm referring to the aromatic hydrogens; **c** ^{1}H NMR (in DMSO-d_6) of CPPMA and **d** its local enlarged drawing from a range of 7.8-6.8 ppm ascribed to the aromatic hydrogens.

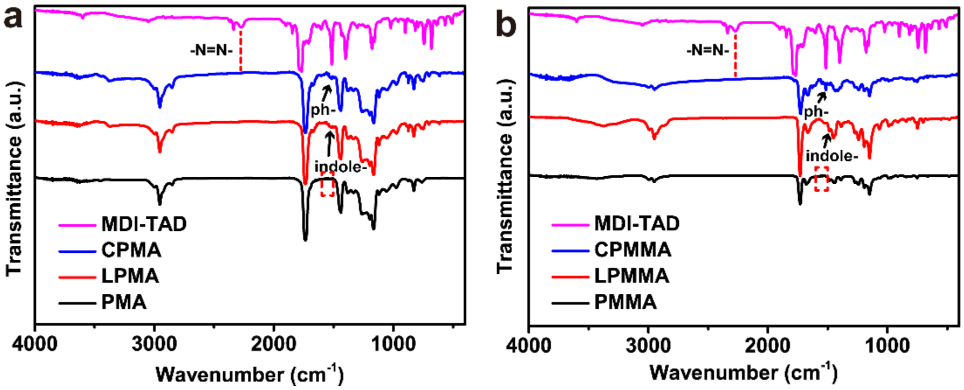

Supplementary Figure 8. Fourier transform infrared spectroscopy of **a** PMA, LPMA, CPMA, MDI-TAD and **b** PMMA, LPMMA, CPMMA, MDI-TAD.

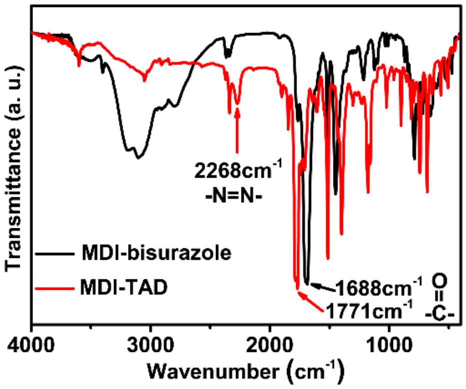

Supplementary Figure 9. Fourier transform infrared spectroscopy of 4,4'-(4,4'-diphenylmethylene)-bis-(urazole) (MDI-bisurazole) and 4,4'-(4,4'-diphenylmethylene)-bis-(1,2,4-triazoline-3,5-dione) (MDI-TAD). Strong absorption peaks at 1688 cm^{-1} and 1771 cm^{-1} can be attributed to the stretching vibration of carbonyl groups.

Supplementary Table 2. Solubility and transparency of related PMA and PMMA. TAD-indole crosslinked polymer (CPMA and CPMMA) tested as insoluble, even when soaked for one month in above solutions at room temperature, while they can be dissolved in DMA, DMSO, DMF and NMP in half an hour at 120 °C. The soluble fraction was tested by calculating the mass difference before and after the films were soaked in DMSO for seven days at ambient temperature.

Sample	DMA[a]	DMSO	DMF[b]	NMP[c]	THF[d]	Transparency	Soluble fraction (wt%)
LPMA	++[e]	++	++	++	++	Light yellow	100
LPMMA	++	++	++	++	++	Light yellow	100
CPMA	+-	+-	+-	+-	--	Yellow	0.86
ir-CPMA	--	--	--	--	--	Yellow	0.64
du-CPMA	--	--	--	--	--	Light yellow	0.68
CPMMA	+-	+-	+-	+-	--	Yellow	0.32

[a]*N*,*N*-Dimethylacetamide (DMA); [b]*N*,*N*-Dimethylformamide (DMF); [c]*N*-Methyl-2-pyrrolidone (NMP); [d]Tetrahydrofuran (THF); [e]++: The polymer can be completely dissolved at room temperature; +–: the polymer can only be dissolved at 120 °C. --: the polymer was insoluble at high and room temperature.

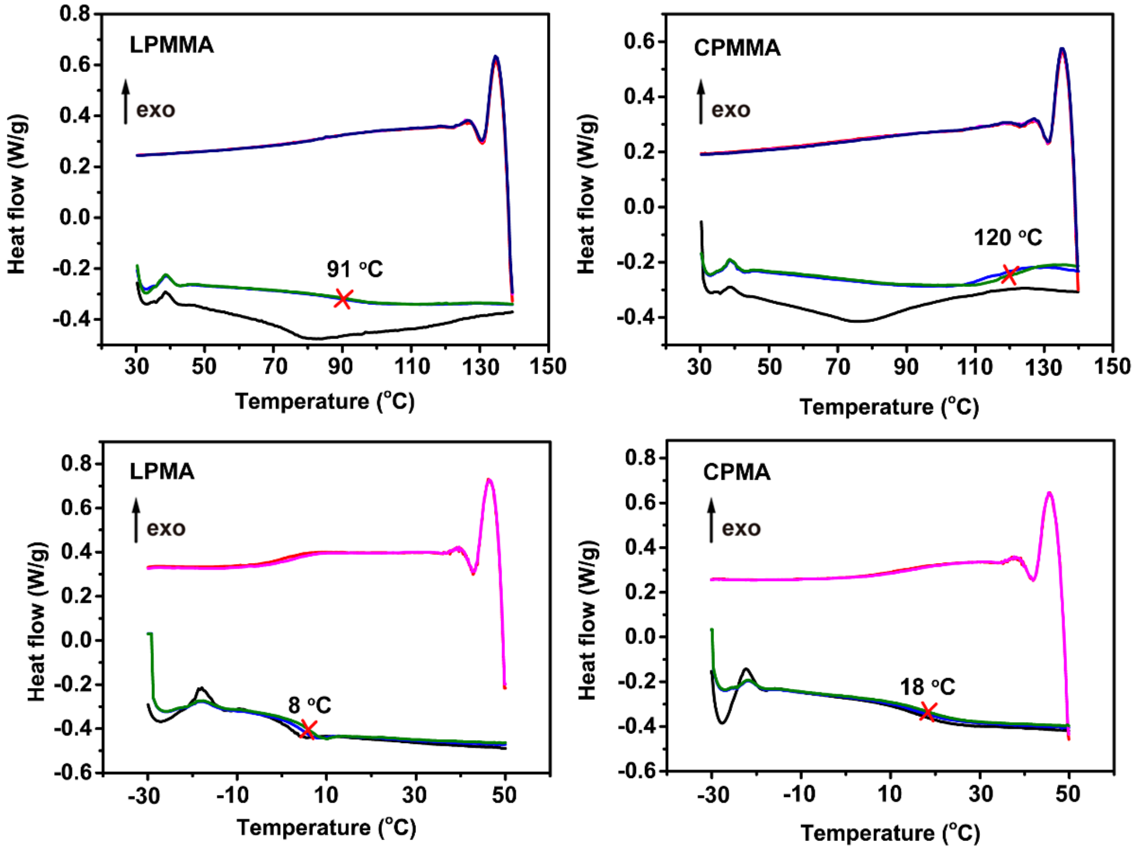

Supplementary Figure 10. Differential scanning calorimetry analysis (DSC) of LPMMA, CPMMA, LPMA and CPMA films.

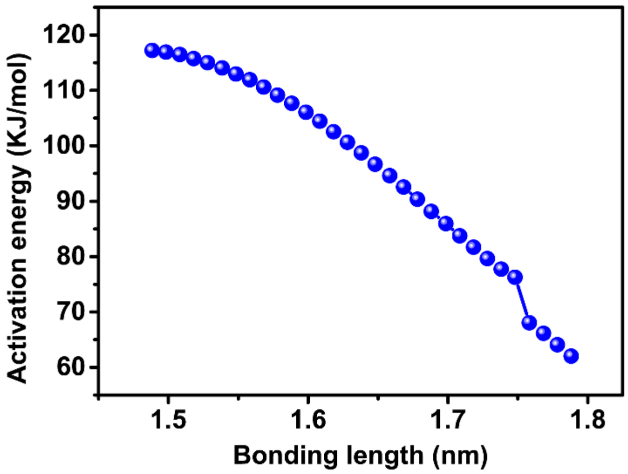

Supplementary Figure 11. Effect of stress on activation energy before the maximum force applied on TAD-indole adducts.

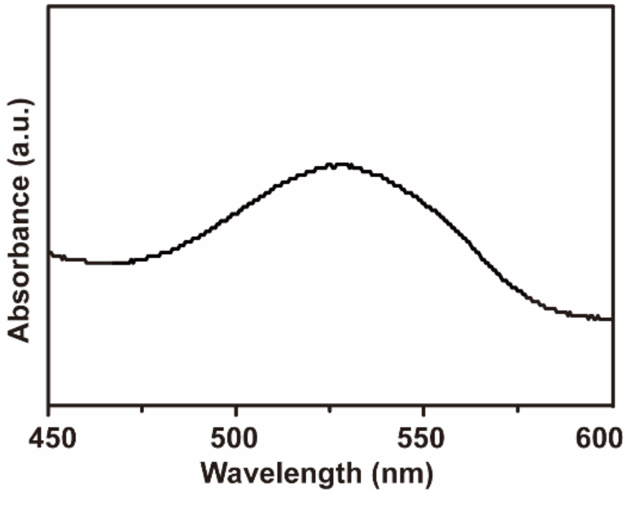

Supplementary Figure 12. UV/vis absorption spectroscopy of MDI-TAD in acetonitrile, indicating the n-π* absorption peak of the TAD azo-bond (-N=N-).

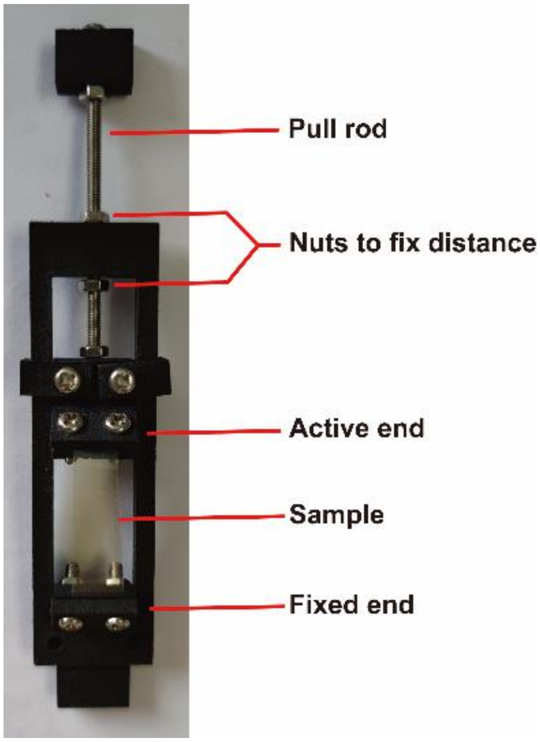

Supplementary Figure 13. A simple relaxation fixture device made by 3D printing used for the *in situ* stress relaxation experiments.

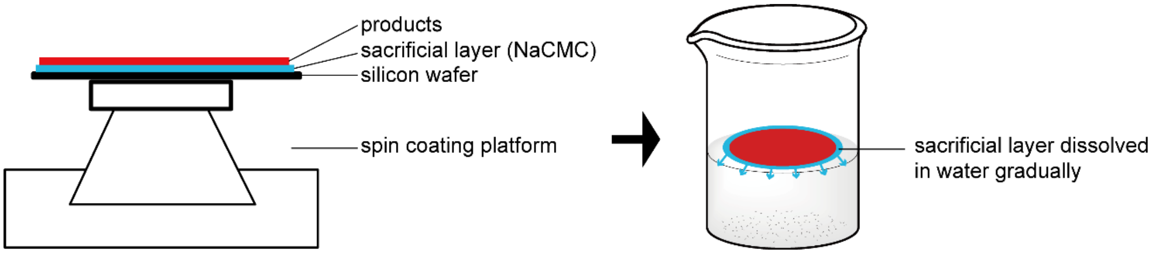

Supplementary Figure 14. Preparation process of spin coated films (CPMA and CPMMA).

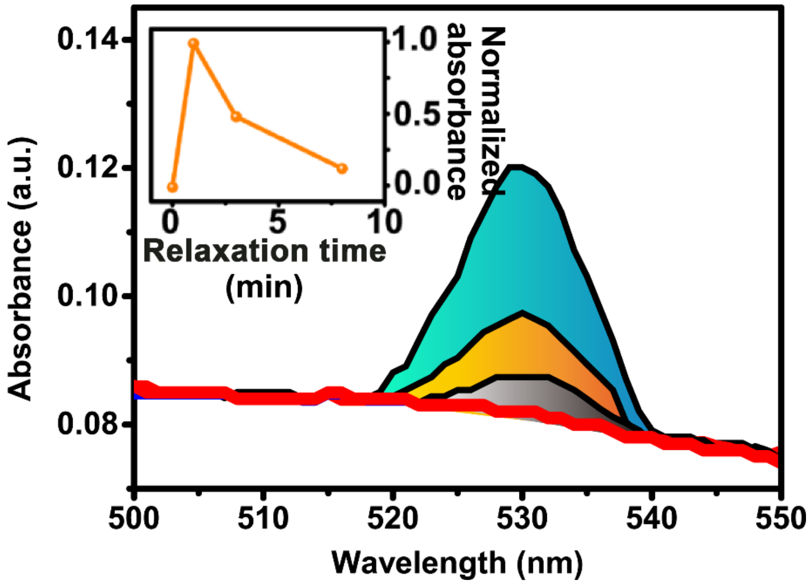

Supplementary Figure 15. The *in situ* stress relaxation-UV/vis spectrum used to characterized the response of the TAD absorption over the stress relaxation time in CPMA film.

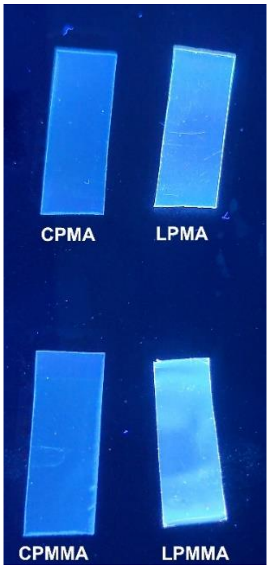

Supplementary Figure 16. Visible fluorescence CPMA, LPMA, CPMMA and LPMMA (λ_{exc} = 365 nm).

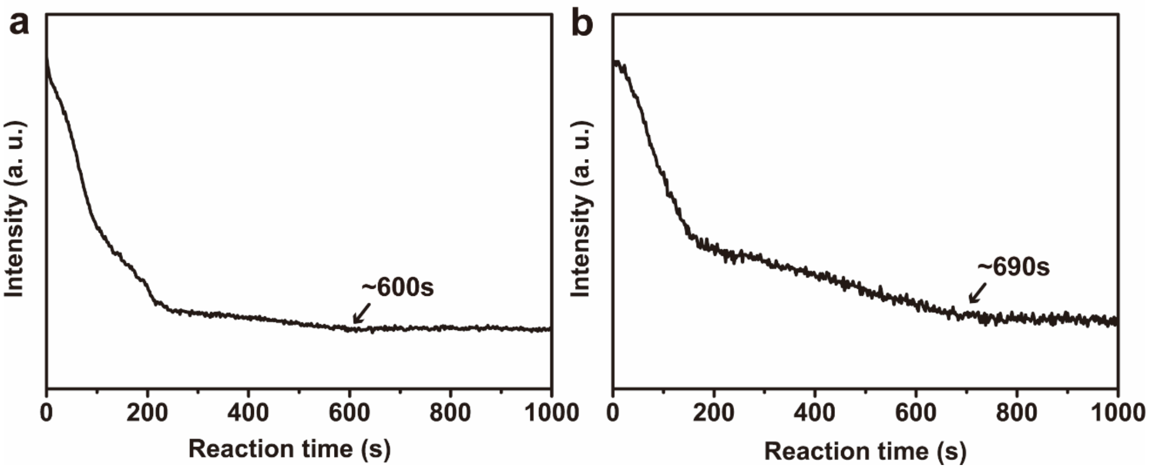

Supplementary Figure 17. Fluorescence intensity changes of **a** CPMA and **b** CPMMA during film formation process at 460 nm, detected under 365 nm excitation by time-resolved fluorescence spectroscopy.

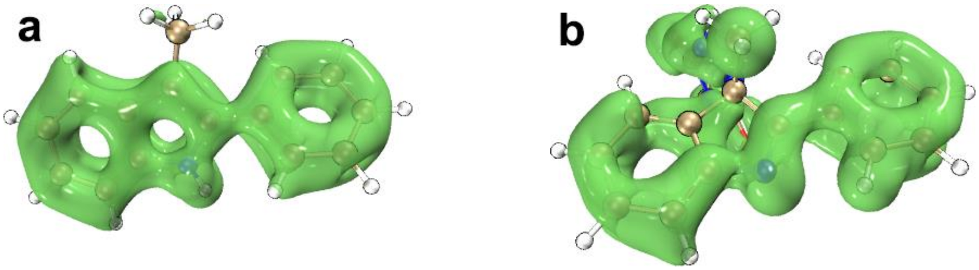

Supplementary Figure 18. Localized orbital locator (LOL) of π electronics isosurfaces for **a** 2-phenylindole and **b** TAD-indole adduct.

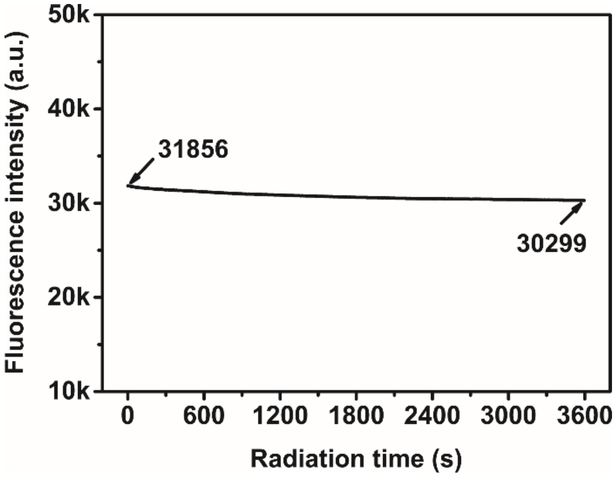

Supplementary Figure 19. The 460 nm fluorescence intensity changes for LPMMA film at 365 nm excitation light irradiation for one hour.

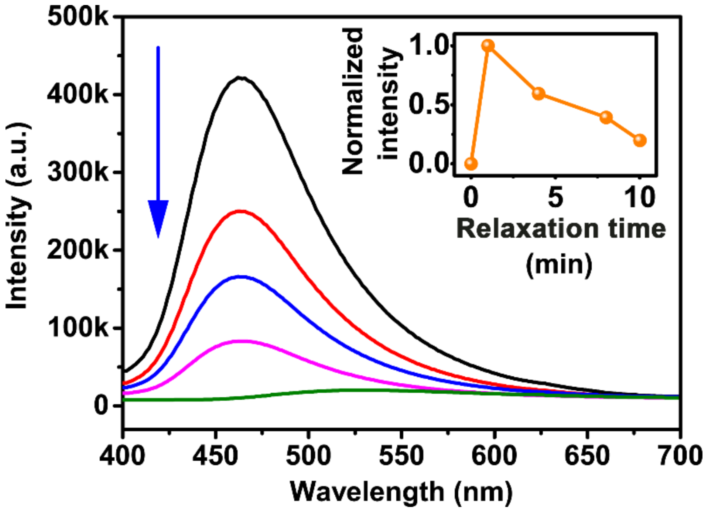

Supplementary Figure 20. The *in situ* stress relaxation-RF spectroscopy equipped with time-resolved program was used for tracking fluorescence intensity changes at 460 nm during relaxation time for CPMA film.

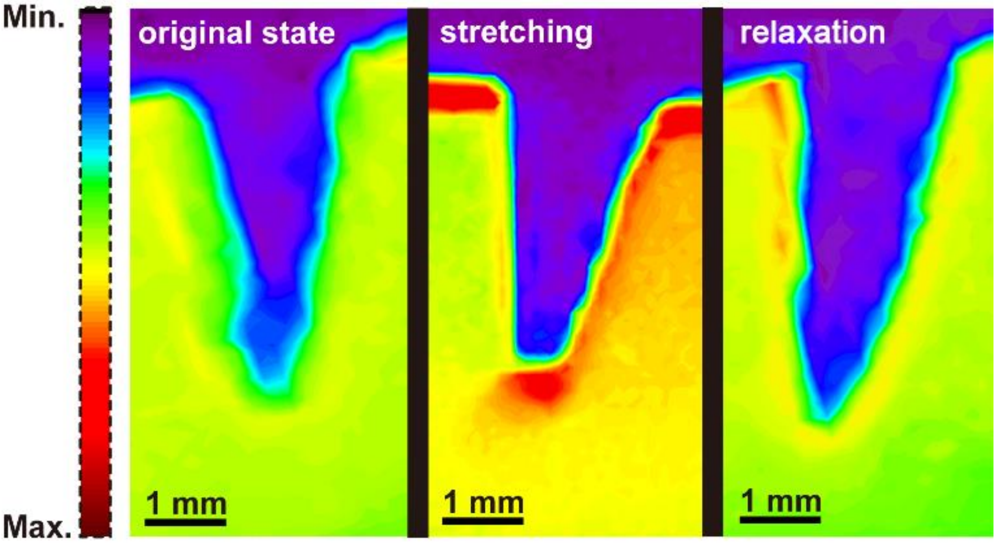

Supplementary Figure 21. Fluorescence mapping to show where C-N bonds break and reform in CPMA film during one cycle.

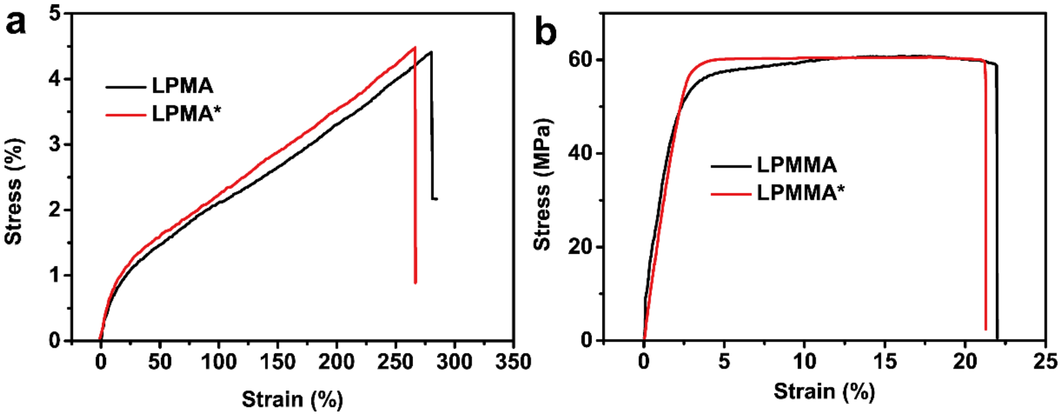

Supplementary Figure 22. Stress as a function of strain of **a** LPMA and LPMA* ($\dot{\varepsilon}$ = 20 mm min^{-1}) and **b** LPMMA and LPMMA* ($\dot{\varepsilon}$ = 2 mm min^{-1}) films. LPMA* and LPMMA*films have the same amount of Ph-TAD reactive with indole (5 mol%) in the polymer network.

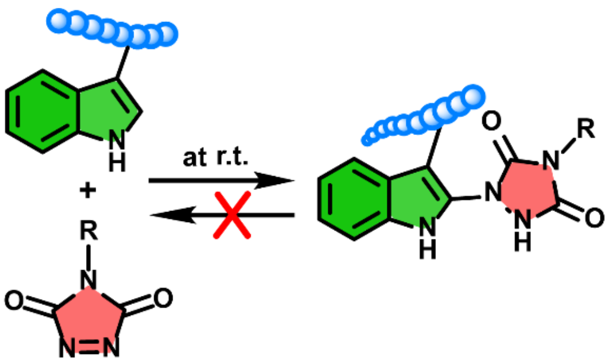

Supplementary Figure 23. Overview of an irreversible TAD-indole click reaction when no substituent on the indole C2-position of NIAM is present. The TAD can rearrange into the indole C2-position to give a more thermodynamically favored adduct with regained aromaticity, thereby increasing the driving force for the TAD-indole adduct to open up.

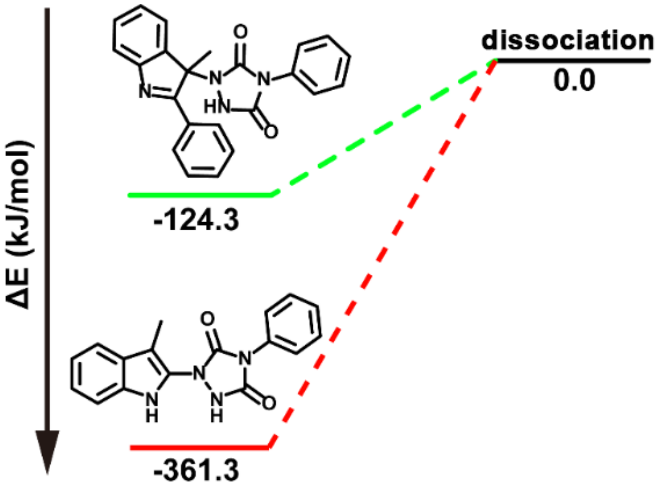

Supplementary Figure 24. Relative enthalpy profiles for TAD-indole adducts formed upon C-N formation in the indole C3- or C2-position, relative to dissociation. For Cartesian coordinates, refer to Supplementary Table 3.

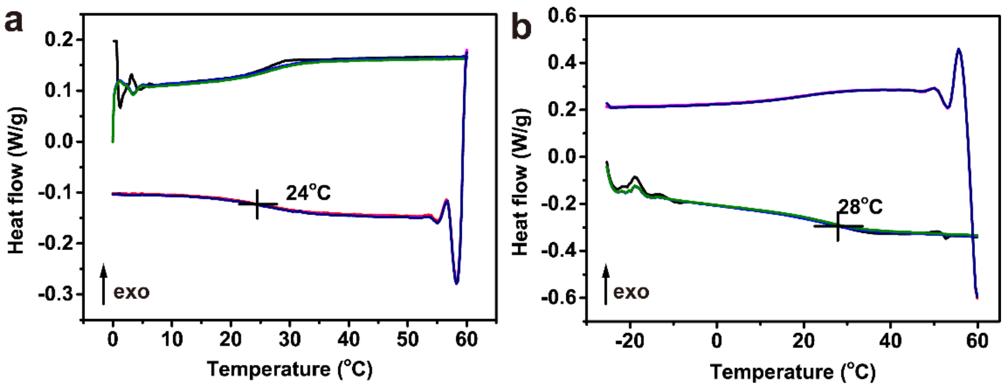

Supplementary Figure 25. Differential scanning calorimetry analysis (DSC) of **a** du-CPMA and **b** ir-CPMA films.

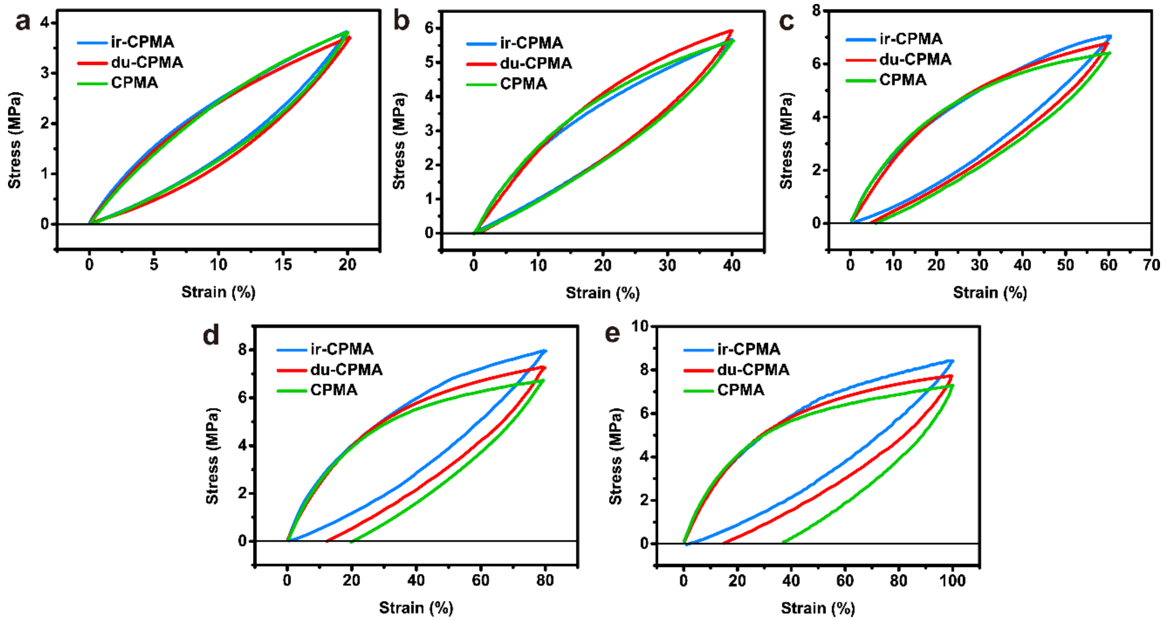

Supplementary Figure 26. One cyclic stretching of different crosslinked films at 40 °C under different stretching strain of **a** 20%, **b** 40%, **c** 60, **d** 80%, **e** 100%

Supplementary Table 3. Cartesian coordinates of the optimized geometry [b3lyp/6-31+G(d,p)] of TAD-indole adduct in the indole C3, C2-position, OI and OI-TS.

TAD-indole adduct in the indole C3-position			
Atom	X	Y	Z
C	-0.26141400	4.32163700	0.05752800
C	-1.03282200	4.45149800	-1.10364100
C	-1.83193500	3.39599200	-1.56149300
C	-1.82666300	2.21093900	-0.82933900
C	-1.05627800	2.07826900	0.33722200
C	-0.27300000	3.12777400	0.79876700
N	-2.54880800	1.03871000	-1.12949400
C	-2.25520300	0.13686000	-0.24707400
C	-1.33528200	0.68866100	0.88525000
C	-2.86443000	-1.20271400	-0.27833400
N	0.69707800	0.23648100	2.20596400
C	-2.13568000	0.76049600	2.20260900
C	2.02710600	-0.07670800	1.90287700
N	2.04497300	-0.38720500	0.54050700
C	0.73875600	-0.38782300	0.01881400
N	-0.12083400	-0.14566900	1.09968600
C	-3.90288900	-1.45603700	-1.19766500
C	-4.50615200	-2.70826000	-1.25976400
C	-4.08681400	-3.73766400	-0.40819000
C	-3.05560200	-3.50157000	0.50263000
C	-2.44873700	-2.24588800	0.56895700
C	3.22392300	-0.67903200	-0.22053100
C	4.22528700	-1.48232800	0.33511700
C	5.37422100	-1.75498500	-0.40928100
C	5.51941000	-1.24211400	-1.70067800
C	4.50990600	-0.44554900	-2.24703200
C	3.36117600	-0.15402600	-1.50989300
O	0.40500900	-0.56234100	-1.13377300
O	2.94796500	-0.07917500	2.69496900
H	0.35110300	5.15338500	0.39239800
H	-1.01138700	5.38573300	-1.65725600
H	-2.43552300	3.48559700	-2.45890700
H	0.32098600	3.03612200	1.70304000
H	-1.57339900	1.31011600	2.96252700
H	-3.06958600	1.29944700	2.02658300
H	-2.38155300	-0.23794900	2.57487500
H	-4.21876500	-0.65421800	-1.85547500
H	-5.30556600	-2.88497000	-1.97356700
H	-4.55827100	-4.71488700	-0.45847000
H	-2.71601500	-4.29682900	1.15988800

H	-1.63135100	-2.08632500	1.26178000
H	4.11425700	-1.87062500	1.33996600
H	6.15448900	-2.37327000	0.02413300
H	4.61380100	-0.04340900	-3.25020800
H	2.57389300	0.45701100	-1.93351900
H	0.38783800	-0.15212800	3.09203700
H	6.41308600	-1.46145100	-2.27716300

OI			
Atom	X	Y	Z
C	-0.76475200	4.39935300	-0.19779700
C	-1.41430200	4.32851000	-1.43648200
C	-2.00473200	3.13598700	-1.87532400
C	-1.90921500	2.04686200	-1.01965200
C	-1.28082900	2.09948600	0.22463400
C	-0.69291600	3.28409700	0.65128900
C	-2.02332600	-0.10461000	-0.24339700
C	-1.36491300	0.72489400	0.88074700
C	-2.51979800	-1.47503500	-0.15741200
N	0.69800300	0.73919000	2.23158700
C	-2.25206300	0.74981900	2.14389600
C	1.97645500	0.54290700	1.87968000
N	1.99045300	-0.14425400	0.54560600
C	0.69880400	-0.25932200	0.13707100
N	-0.06570800	0.15058300	1.19934800
C	-3.61619700	-1.89312600	-0.93870400
C	-4.08615400	-3.20103100	-0.85603800
C	-3.47300400	-4.11053100	0.01124100
C	-2.39308100	-3.70274700	0.80006100
C	-1.91846200	-2.39661000	0.72244200
C	3.14118400	-0.55232900	-0.18332500
C	4.31647200	-0.88555800	0.50718400
C	5.44464900	-1.29006600	-0.20774500
C	5.41792600	-1.37364700	-1.60247500
C	4.24444200	-1.04136800	-2.28347000
C	3.10916700	-0.62631000	-1.58481000
O	0.18831100	-0.63988300	-0.94500800
O	3.01502600	0.82617200	2.47401000
H	-0.30363900	5.33268600	0.10944500
H	-1.45618900	5.20684900	-2.07275000
H	-2.50095800	3.07262200	-2.83897200
H	-0.17530800	3.32739900	1.60346200
H	-1.73843800	1.35259900	2.89530700
H	-3.22804700	1.19115200	1.92395700
H	-2.38709700	-0.25883700	2.53966700
H	-4.13327700	-1.19460700	-1.59030700

H	-4.93516200	-3.50732700	-1.45897200
H	-3.83954200	-5.13058200	0.07531700
H	-1.91374500	-4.40570700	1.47383800
H	-1.06921100	-2.08586100	1.31953400
H	4.34071800	-0.79558300	1.58517100
H	6.35120500	-1.54140800	0.33563800
H	4.20734100	-1.09955900	-3.36787400
H	2.19896800	-0.37690700	-2.11529700
N	-2.38729500	0.72652400	-1.21215000
H	-2.80455200	0.41222700	-2.07854700
H	6.29993200	-1.69152300	-2.15105400

OI-TS			
Atom	X	Y	Z
C	-0.80025200	4.39563900	-0.19113600
C	-1.44244300	4.32408600	-1.43395200
C	-2.02859700	3.13085500	-1.87588700
C	-1.93701900	2.04203300	-1.01915900
C	-1.31806800	2.09584400	0.22900400
C	-0.73306500	3.28092400	0.65864000
C	-2.04440000	-0.10898000	-0.24597900
C	-1.39896100	0.72157400	0.88658100
C	-2.49529900	-1.49313500	-0.16791400
N	0.66688700	0.72103900	2.23555400
C	-2.28241900	0.72159400	2.17532500
C	1.94895700	0.53278500	1.88939100
N	1.97391000	-0.12512600	0.54433800
C	0.68435700	-0.23318800	0.12108000
N	-0.08699000	0.15737700	1.18478100
C	-3.45960300	-1.99808000	-1.06411400
C	-3.87734000	-3.32258700	-0.98128400
C	-3.34319100	-4.16483600	-0.00007700
C	-2.39423500	-3.67260300	0.90018900
C	-1.97316200	-2.34792000	0.82353700
C	3.13056400	-0.52159700	-0.18147300
C	4.29899800	-0.87075400	0.51287100
C	5.43295900	-1.26336600	-0.19953300
C	5.41889400	-1.31942800	-1.59583600
C	4.25222400	-0.97141400	-2.28071700
C	3.11135700	-0.56794400	-1.58440500
O	0.18728800	-0.59655100	-0.97203700
O	2.98125900	0.80371800	2.50069100
H	-0.34203500	5.32951800	0.11882700
H	-1.48157900	5.20231100	-2.07052100
H	-2.51780700	3.06618900	-2.84304500
H	-0.22327100	3.32529400	1.61524100

H	-2.57649700	1.74000500	2.43200700
H	-3.18750900	0.12220300	2.05235500
H	-1.69011600	0.32002300	2.99898100
H	-3.90958500	-1.36050700	-1.81965300
H	-4.62254300	-3.69674100	-1.67609100
H	-3.66933400	-5.19854600	0.06317500
H	-1.97584900	-4.32276100	1.66183100
H	-1.22127900	-1.97257300	1.50798900
H	4.31374400	-0.80200900	1.59258900
H	6.33405400	-1.52711200	0.34708400
H	4.22491400	-1.00811100	-3.36637100
H	2.20643900	-0.30654900	-2.11809900
N	-2.41022200	0.72032700	-1.21466200
H	-2.82206300	0.40783100	-2.08395900
H	6.30531300	-1.62813500	-2.14254900

TAD-indole adduct in the indole C2-position			
Atom	X	Y	Z
C	-5.63311600	0.34101000	-1.38633400
C	-5.81456500	-0.74093200	-0.50585800
C	-4.78802200	-1.17199000	0.31493800
C	-3.55344100	-0.51198400	0.25815000
C	-3.38989600	0.56612400	-0.64160400
C	-4.42426400	1.01040200	-1.46601500
C	-2.31637300	-0.67733200	0.97888700
C	-1.48139100	0.27220600	0.47576300
N	-2.09655000	1.02005400	-0.51095900
C	-2.00034200	-1.69539700	2.02368000
N	-0.13195000	0.50558100	0.80540600
N	0.34087100	1.82757900	0.63343800
C	1.72320000	1.75581100	0.40045800
N	2.00536000	0.40995200	0.18892300
C	0.86667300	-0.38071200	0.38866700
O	0.75224100	-1.56175100	0.23803700
O	2.47836600	2.68596200	0.39959400
C	3.29006900	-0.10429900	-0.16690600
C	3.37997600	-1.12421900	-1.10954400
C	4.63139600	-1.62385600	-1.44783100
C	5.77990400	-1.10258000	-0.86173500
C	5.67555200	-0.07927000	0.07413800
C	4.43028800	0.42215400	0.43271600
H	-6.45562600	0.65557100	-2.01723200
H	-6.77478600	-1.24102200	-0.47396200
H	-4.93269900	-2.00654800	0.99172900
H	-4.28528400	1.84149200	-2.14732500

H	-1.68443600	1.82243100	-0.95906500
H	-1.07872900	-1.43611900	2.54672000
H	-2.80958200	-1.76288600	2.75453600
H	-1.85722900	-2.68252800	1.57774700
H	0.10734100	2.43158200	1.41626600
H	2.48170300	-1.52799100	-1.55645500
H	4.70558100	-2.42176900	-2.17642500
H	6.75317100	-1.49262600	-1.13351000
H	6.56602000	0.33209500	0.53337800
H	4.34168800	1.22165400	1.15547500
